# Lao Noma Survivors: A Case Series, 2002–2020

**DOI:** 10.4269/ajtmh.21-1079

**Published:** 2022-02-28

**Authors:** Margaret Leila Srour, Elise Farley, Emmanuel Kabengele Mpinga

**Affiliations:** ^1^ Health Frontiers, Vientiane, Laos;; ^2^ Nudibrink Research Consultancy, Cape Town, South Africa;; ^3^ University of Geneva, Geneva, Switzerland

## Abstract

Noma is a rapidly progressing infection of the oral cavity, mostly affecting children aged between 2 and 5 years. If untreated, mortality can reach 90% within a few weeks after the onset of symptoms. Most of the published literature on noma are case reports or case series from Africa. Studies including noma survivors in Asia are limited. We present a case series of noma survivors in Laos. A retrospective analysis of data collected to monitor the care provided to Lao noma survivors who presented for treatment from 2002 to 2020 was conducted. The review assessed data including sociodemographic characteristics, diagnosis, mouth opening, self-reported quality of life at admission and after surgery, and the names used for the disease. Of the 50 patients included, 25 (50%) were female. The median age of self-reported onset of acute noma was 4 years (interquartile range [IQR] 2–7 years). The noma survivors came from 14/17 (82%) of Lao provinces. There were 64 surgeries conducted on 45 of these survivors. There was a median of 25 years (IQR 16–33 years) between the time of acute infection and the provision of surgical care. Improvements in nutritional status and quality of life were evident after surgery. Patients referred to the disease as “Pak Phuey,” which means diseased mouth. Noma survivors frequently live for years with disabling sequelae. Surgical rehabilitation improves the quality of life for noma survivors.

## INTRODUCTION

Noma is a rapidly progressing infection of the oral cavity, mostly affecting children aged between 2 and 5 years. ^1^ The first stages of noma are simple and acute necrotizing gingivitis, rapidly progressing to edema, necrosis, gangrene, scarring, and sequelae. If untreated, noma has an estimated 90% mortality rate. ^1^^,^ ^2^ Treatment of acute noma with antibiotics, nutritional support, wound debridement, and treatment of underlying conditions can greatly reduce morbidity and mortality. ^1^ The prevention of acute noma focuses on eliminating the known risk factors for the disease, including poor living conditions, chronic malnutrition, limited access to healthcare including immunizations, and poor oral hygiene practices. ^3^ If these risk factors are eliminated, noma can be eradicated. ^4^ Noma survivors suffer from functional and cosmetic problems, often leading to social exclusion and lack of opportunities from a young age. ^3^^,^ ^5^ Surgical rehabilitation for these patients is challenging, expensive, and often difficult to access or unavailable in endemic countries. ^6^

The majority of published literature on noma are case reports, case series, or retrospective studies, mostly from low-income countries in Africa. ^7^^–^ ^13^ Currently, the WHO, noma control program only operates in Africa. ^14^ Current studies, including noma survivors in Asia, are limited. Few reports of surgical rehabilitation include follow-up after 1 year. Many publications are from the 1800 and 1900s. ^15^^–^ ^24^ Prior to 2008, noma was not reported in Laos in the scientific literature. ^25^ We present a case series of 50 Lao noma survivors who presented for care between 2002 and 2020, with post-surgical follow-up at least 1 year after surgery, in the majority of cases. This article aims to report about noma outside Africa to encourage education, research, and case findings in countries with noma risk factors.

## MATERIALS AND METHODS

We conducted a retrospective review of data collected to monitor patient care from noma patients who presented for care in Laos, primarily at Mahosot Hospital, Vientiane, by Bridge the Gap (Dutch surgical organization) between 2006 and 2020. All patients gave written informed consent for the description of their clinical details and photographs, and the Declaration of Helsinki has been followed. The patients and guardians, in the case of minors, were informed about noma, shown published photos in medical journals of noma patients, and were asked permission to share their history and photos in the medical literature. Because of the limited number of recent reports of noma cases in Asia, it was decided to conduct a retrospective review of the data. This data included sociodemographic characteristics, information on the patients’ acute illness, mouth opening before and approximately 1 year after surgery, the survivors’ quality of life prior to care being sought and after surgery, and the names that survivors and their families used to describe the disease. This research fulfilled the exemption criteria set by the Lao National Ethics Committee for Health Research for *a posteriori* analysis of routinely collected clinical data and thus did not require full ethical review. It was conducted with permission from the Lao National Ethics Committee for Health Research, recorded No. 047/NECHR.

## DATA ANALYSIS

We performed a descriptive analysis. Categorical variables are reported as frequencies and percentages. Continuous variables are summarized using means and standard deviations (SD), or medians and interquartile ranges (IQRs), depending on normality. Missing data numbers are recorded in each table. All analyses were conducted in Stata 15 (StataCorp LLC 2017, Stata Statistical Software Release 15, College Station, TX).

## RESULTS

**Figure 1.**
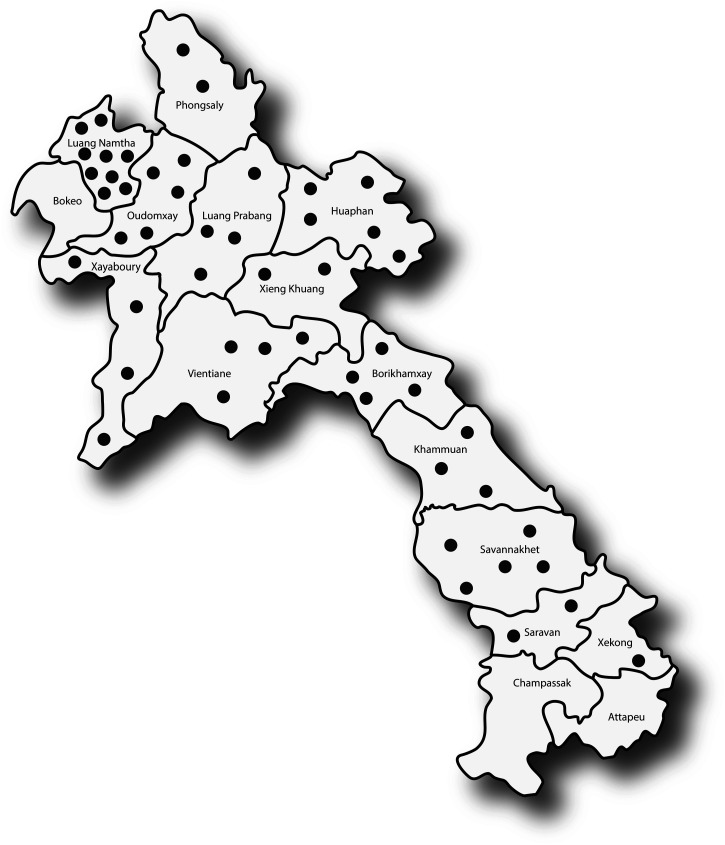
Map of the home villages of Laos noma survivors included in the study cohort. *Please note that this map does not represent the distribution of noma cases around the country. It only represents the survivors included in the study cohort. These limitations should be taken into consideration when interpreting the information portrayed in this map.

All the noma patients (*N* = 50) who presented for care from 2002 to 2020 were included in the study. Half (*N* = 25, 50%) were females. The survivors came from 14/17 (82%) Lao provinces. One province, Luang Namtha, is the home of 9/50 (18%) noma survivors in the cohort, and one district, Nalae, is home to four (8%) survivors (Figure 1). Of the respondents, 19 (38%) were Lao Lum (the majority [68%] of Lao inhabitants belong to the Lao Lum group), and 28 (56%) were ethnic minorities; the rest were unknown. The year of disease onset ranged from 1960 to 2005. The median age of self-reported onset of acute noma was 4 years (IQR 2–7 years) (Table 1).

**Table 1:** Lao noma survivors—sociodemographic characteristics, clinical presentation, quality of life before surgery, and medical care received

		*N* = 50	%
Gender	Male	25	50
	Female	25	50
Province	Luang Namtha	9	18
	HuaPhan	5	10
	Oudomxay	5	10
	Savannakhet	5	10
	Luang Prabang	4	8
	Sayabouly	4	8
	Bolikhamsai	4	8
	Khammouane	3	6
	Phongsali	2	4
	Vientiane	2	4
	Xaisomboun	2	4
	Xieng Khouang	2	4
	Salavan	2	4
	Sekong	1	2
Ethnicity	Lao Lum	19	38
	Khamu	10	20
	Other	18	36
	Missing	3	6
Year of onset	1960–1979	10	20
	1980–1999	31	62
	2000–2005	6	12
	Missing	3	6
Age at self-reported onset of symptoms (median, IQR)	4 years	2–7 y	2–7 y
Mouth opening compromised	Yes	21	42
	No	27	54
	Missing	2	4
Ankylosis, complete inability to open or close the mouth	Yes	13	26
	No	35	70
	Missing	2	4
Eating difficulty before surgery (self-reported)	Yes	20	40
	No	27	54
	Missing	3	6
Speaking difficulty before surgery (self-reported)	Yes	19	38
	No	28	56
	Missing	3	6
Poor cosmetic appearance before surgery (self-reported)	Yes	44	88
	No	4	8
	Missing	2	4
Attend school before surgery (self-reported)	Yes	21	42
	No	26	52
	Missing	3	6
Able to marry before surgery (self-reported)	Yes	24	48
	No	19	38
	NA	4	8
	Missing	3	6
Number of years from acute infection to provision of surgical care (median, IQR) (*N* = 47)	Median 25 years	16, 33 years	16, 33 years
Number of surgeries	0	4	8
	1	30	60
	2	8	16
	3	6	12
	Missing	2	4

IQR = interquartile range.

Before surgery, all (*N* = 50; 100%) of the noma survivors reported functional problems, cosmetic problems, or both because of noma sequelae. Survivors (*N* = 21; 42%) experienced problems with mouth opening, and 13 (26%) had complete ankylosis (inability to open or close their mouths). Compromised mouth opening made speaking (*N* = 19; 38%) and eating (*N* = 20; 40%) difficult. Most of the patients (*N* = 44; 88%) were unhappy with their appearance. The majority of survivors (*N* = 26; 52%) had never been to school. Some survivors (*N* = 19; 38%) of marriageable age reported they were unable to marry because of their appearance and hoped that surgery would improve their marital prospects (Table 1).

Some (*N* = 19; 38%) received basic medical care at the time of their acute disease. There was a median of 25 years (IQR 16–33 years), with a range of 5–55 years between the time of acute infection and the provision of surgical care. A total of 64 surgeries were conducted on 45 survivors. The majority (*N* = 30; 60%) of patients had one surgery. A small number (*N* = 4; 8%) patients were declined for surgery for safety reasons.

Most (29/45; 65%) patients who had surgery were followed up after surgery. The median time of follow-up was 2 years (IQR 1–3 years) (Table 2). The majority of patients (*N* = 21; 72%) had one assessment, and eight (28%) had two assessments (as they had surgery after the first follow-up visit). All the patients were encouraged to return, including reimbursement of travel costs, for follow-up in 1 year. As much as possible, they were contacted to remind them to return. The patients who did not return for follow-up may have been satisfied or unsatisfied with the results or the long journey, often 1–3 days, to the capital city may not have been possible.

**Table 2:** Lao noma survivors—self-reported quality of life after surgery

		*N* = 29	%
Mean follow-up time (*N* = 27)	Mean 2 years	IQR 1–3 years	
Appearance after surgery	Much better	17	59
	Better	10	34
	Worse	1	3
	Much worse	1	3
	Missing	0	0
Speech after surgery	Better	20	69
	Same	5	17
	Worse	3	10
	Missing	1	3
Eating after surgery	Easier	22	76
	Same	4	14
	Worse	2	7
	Missing	1	3
Weight after surgery	More	18	62
	Same	6	21
	Less	1	3
	Unknown	3	10
	Missing	1	3
Marital status after surgery	Married before surgery	13	45
	Married after surgery	4	14
	Single	12	41
	Missing	0	0
Ease of life after surgery	Better	22	76
	Same	3	10
	Worse	2	7
	Missing	2	7
Treatment by others after surgery	Better	21	72
	Same	4	14
	Worse	1	3
	Missing	3	10
Questions about face by others after surgery	Less	18	62
	Same	4	14
	More	5	17
	Missing	2	7
Shyness after surgery	Less	7	24
	Same	1	3
	More	2	7
	Not shy	17	59
	Missing	2	7
Frequency of leaving village after surgery	More	20	69
	Same	7	24
	Missing	2	7
Face covering worn after surgery	Stopped after surgery	4	14
	Less	7	24
	Same	3	10
	Never used	13	45
	Missing	2	7
Current problems	Cosmetic	4	14
	Functional	10	34
	Pain	1	3
	None	12	41
	Missing	2	7
Glad had surgery	Yes	29	100
	Missing	0	0
Further surgery	Yes—look better	12	41
	Yes—improve function	3	10
	Yes—stop pain	1	3
	No need	9	31
	No—afraid of further surgery	3	10
	Missing	1	3

IQR = interquartile range.

One (3%) of these patients had a 4-cm improvement in his mouth opening, five (17%) had a 2-cm improvement, and eight (28%) had a 1-cm improvement in mouth opening.

**Figure 2.**
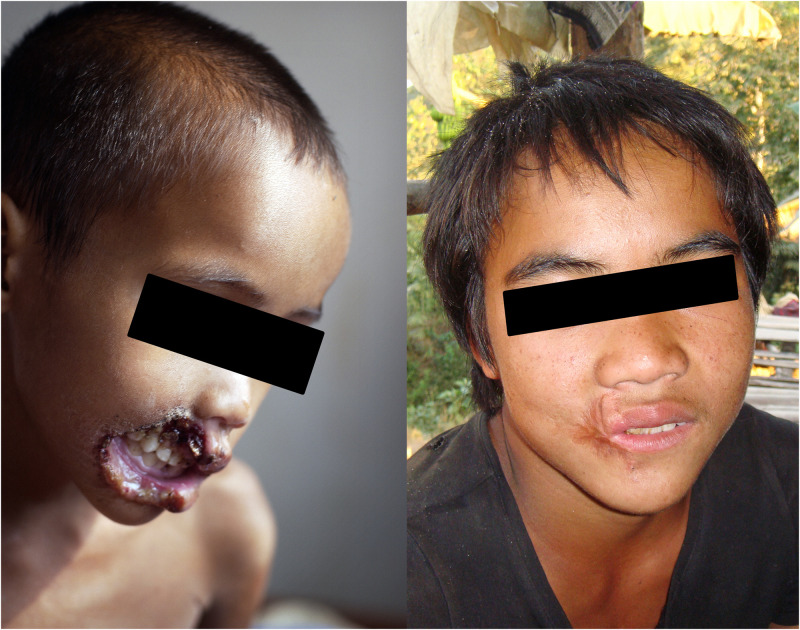
Five-year-old boy, acute disease and after surgery. This figure appears in color at www.ajtmh.org.

In 2003, a 5-year-old boy was identified in a remote district hospital with acute noma. He was treated with antibiotics and nutritional support. The parents took him home and the doctors believed that he had died. Several years later, he was found in a mountain village. He suffered with oral incontinence and was extremely shy. Surgical rehabilitation with two surgeries 1 year apart resulted in resolution of the incontinence (Figure 2).

All 29 patients who were seen after surgery reported that they were glad to have had surgery, and several functional improvements were self-reported. These included improvements in weight (*N* = 18; 62%), appearance (*N* = 27; 93%), speech (*N* = 20; 69%), and eating (*N* = 22; 76%). Survivors reported that the surgery had improved their quality of life (*N* = 22; 76%), they were treated better by others (*N* = 21; 72%), and asked fewer questions about their face (*N* = 18; 62%). After surgical treatment, four (14%) survivors got married. These four attributed their ability to marry to their surgical treatment (Table 2).

**Figure 3.**
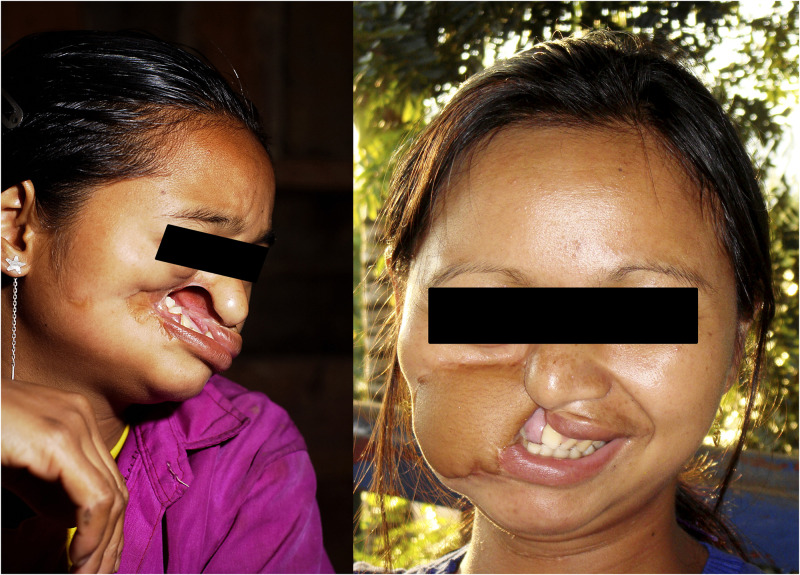
Sixteen-year-old young woman, before and after surgery. This figure appears in color at www.ajtmh.org.

The first reported Lao noma survivor, a 16-year-old female, was identified in 2002 in a poor mountain village. She had acute noma at 4 years of age and received some medical care at a district hospital. The sequelae of noma resulted in a large hole in her face and ankylosis, which caused her to be unable to open and close her mouth. She had difficulty speaking and eating, requiring pushing food with her fingers up against her teeth. This patient was very shy and reluctant to socialize even in her small village. Surgical rehabilitation was challenging and performed in Singapore. ^26^ She returned to her village, got married, and had three children. Her shyness resolved allowing her to socialize within and outside her village (Figure 3).

The patients and guardians were asked whether they knew the name of this disease and what caused it. Some patients used the name “Pak Phuey,” which means diseased mouth, and “Mob Yeeg,” meaning a disease caused by evil spirits. Others reported that the disease was caused by food poisoning.

## DISCUSSION

Our study describes the characteristics of a cohort of Lao noma survivors. Most patients were aged between 2 and 7 years at the onset of symptoms, a finding corroborated by many other noma studies. ^1^^,^ ^3^^,^ ^27^ Survivors came from almost every province in the country. Most patients reported improvements in their quality of life after surgery, including increased acceptance in their communities and the ability to marry. These findings are mirrored in two Ethiopian studies ^28^^,^ ^29^ and a Nigerian study. ^5^

Our study has shown that the survivors of noma in Laos waited decades before being surgically treated, a finding similar to an Ethiopian study in which the median time from onset of symptoms to access to surgical care was 18 years. ^28^ These decades of suffering from facial disfigurement during childhood and adolescence frequently lead to stigma resulting in social isolation, excessive questions about the survivors’ physical appearance, bullying, and self-consciousness. ^30^ To minimize these harmful effects, noma action plans need to include efforts to reach hidden patients, offer support, and surgical rehabilitation. ^29^

Noma can be prevented by addressing the risk factors including improving prenatal care, promotion of breastfeeding, immunizations, clean drinking water, improved sanitation, and the elimination of extreme poverty. ^2^^,^ ^4^ Early detection of children at risk for noma should be conducted with detection and prevention of malnutrition and routine mouth examination. ^31^ Most healthcare workers and parents do not know about noma, so information campaigns to raise awareness about noma, the recognition of oral ulcers, and early lesions of noma are needed in countries with noma risk factors. ^14^ Training healthcare workers to examine children’s mouths, to recognize gingivitis, and to know when to refer patients could improve early detection of noma. ^32^

The integration of noma surveillance into existing health information systems, health structures, programs such as primary healthcare, vaccination campaigns, malnutrition surveys, and surveillance for other neglected diseases could lead to prevention, early treatment, and case finding of survivors in need of surgical treatment. To aid these efforts, it would be highly beneficial for noma to be included in the World Health Organization’s list of neglected tropical diseases. As noma affects the poorest children in the world, results in high morbidity and mortality, causes stigma and discrimination, and can be prevented with implementation of basic public health measures, noma meets the WHO NTDs criteria for inclusion. ^33^ Inclusion would raise awareness about noma, encourage research and interventions to prevent noma morbidity and mortality, and identify survivors to offer surgical rehabilitation.

There were several limitations to our study. The survivors of noma in Lao do not include the children who died without a diagnosis or survivors unaware or afraid of surgical treatment, suffering from severe stigma or unable to travel for surgical care to the capital city. The patients were identified primarily when they came to the capital city in response to posters or social media offering free treatment, so they do not represent the true prevalence. Many of the noma survivors in this cohort presented decades after their acute illness, suggesting that acute noma may not be a current problem in Lao. However, this cohort includes cases of acute noma in the 21st century (6/50; 12%). The persistence of noma risk factors should encourage public health attention and health worker education about this disease. As with all retrospective reviews of routinely collected data, the amount and type of data collected is limited. No control group was included, and generalizing findings is difficult as a convenience sampling methodology was used. ^34^^–^ ^36^ To reduce these limitations, a prospective study of noma patients with planned follow-up using standardized interviews focusing on the quality of life should be used by organizations treating noma around the world. ^5^^,^ ^28^

## CONCLUSION

The survivors of noma in this report have remained hidden for decades and may represent just a small number of the cases, as untreated noma has a high mortality and poor children’s deaths and diagnoses are not recorded. In this cohort, patients are from almost every province in the country, suggesting noma endemicity. The name for noma, “Pak Phuey,” diseased mouth, indicates local awareness of this disease. The persistence of noma risk factors in Lao implies that noma is still a threat to young children’s lives.

Most noma survivors in Laos presented decades after their acute illness. Surgical rehabilitation did improve the quality of their lives, indicating the need for noma action plans with efforts to reach hidden patients, offer support, and where appropriate, surgical rehabilitation. Noma exists where children are malnourished, without adequate access to healthcare or immunizations and living in poverty. Ultimately, the eradication of this preventable childhood disease should be the goal, requiring national and international attention.
